# Genomic Analysis of *Salmonella enterica* Serovar Typhimurium Characterizes Strain Diversity for Recent U.S. Salmonellosis Cases and Identifies Mutations Linked to Loss of Fitness under Nitrosative and Oxidative Stress

**DOI:** 10.1128/mBio.00154-16

**Published:** 2016-03-08

**Authors:** Hillary S. Hayden, Susana Matamouros, Kyle R. Hager, Mitchell J. Brittnacher, Laurence Rohmer, Matthew C. Radey, Eli J. Weiss, Katie B. Kim, Michael A. Jacobs, Elizabeth H. Sims-Day, Min Yue, Mussaret B. Zaidi, Dieter M. Schifferli, Shannon D. Manning, Judd L. Walson, Samuel I. Miller

**Affiliations:** a Department of Microbiology, University of Washington, Seattle, Washington, USA; b Department of Pathobiology, School of Veterinary Medicine, University of Pennsylvania, Philadelphia, Pennsylvania, USA; c Microbiology Research Laboratory, Hospital General O’Horan, Mérida, Yucatán, Mexico; d Infectious Diseases Research Unit, Hospital Regional de Alta Especialidad de la Penïnsula de Yucatán Merida, Mérida, Yucatán, Mexico; e Department of Microbiology and Molecular Genetics, Michigan State University, East Lansing, Michigan, USA; f Department of Epidemiology, University of Washington, Seattle, Washington, USA; g Department of Pediatrics, University of Washington, Seattle, Washington, USA; h Department of Global Health, University of Washington, Seattle, Washington, USA; i Department of Medicine, University of Washington, Seattle, Washington, USA; j Department of Genome Sciences, University of Washington, Seattle, Washington, USA

## Abstract

*Salmonella enterica* serovar Typhimurium is one of the most common *S. enterica* serovars associated with U.S. foodborne outbreaks. *S*. Typhimurium bacteria isolated from humans exhibit wide-ranging virulence phenotypes in inbred mice, leading to speculation that some strains are more virulent in nature. However, it is unclear whether increased virulence in humans is related to organism characteristics or initial treatment failure due to antibiotic resistance. Strain diversity and genetic factors contributing to differential human pathogenicity remain poorly understood. We reconstructed phylogeny, resolved genetic population structure, determined gene content and nucleotide variants, and conducted targeted phenotyping assays for *S*. Typhimurium strains collected between 1946 and 2012 from humans and animals in the United States and abroad. Strains from recent U.S. salmonellosis cases were associated with five *S*. Typhimurium lineages distributed within three phylogenetic clades, which are not restricted by geography, year of acquisition, or host. Notably, two U.S. strains and four Mexican strains are more closely related to strains associated with human immunodeficiency virus (HIV)-infected individuals in sub-Saharan Africa than to other North American strains. Phenotyping studies linked variants specific to these strains in *hmpA* and *katE* to loss of fitness under nitrosative and oxidative stress, respectively. These results suggest that U.S. salmonellosis is caused by diverse *S*. Typhimurium strains circulating worldwide. One lineage has mutations in genes affecting fitness related to innate immune system strategies for fighting pathogens and may be adapting to immunocompromised humans by a reduction in virulence capability, possibly due to a lack of selection for its maintenance as a result of the worldwide HIV epidemic.

## IMPORTANCE

Nontyphoidal *Salmonella* bacteria cause an estimated 1.2 million illnesses annually in the United States, 80 million globally, due to ingestion of contaminated food or water. *Salmonella* Typhimurium is one of the most common serovars associated with foodborne illness, causing self-limiting gastroenteritis and, in approximately 5% of infected patients, systemic infection. Although some *S*. Typhimurium strains are speculated to be more virulent than others, it is unknown how strain diversity and genetic factors contribute to differential human pathogenicity. Ours is the first study to examine the diversity of *S*. Typhimurium associated with recent cases of U.S. salmonellosis and to provide some initial correlation between observed genotypes and phenotypes. Definition of specific *S*. Typhimurium lineages based on such phenotype/genotype correlations may identify strains with greater capability of associating with specific food sources, allowing outbreaks to be more quickly identified. Additionally, defining simple correlates of pathogenesis may have predictive value for patient outcome.

## INTRODUCTION

The Gram-negative bacterial species *Salmonella enterica* is one of the most common human and animal pathogens worldwide (1–3). Of the six described *S. enterica* subspecies, *S. enterica* subsp. *enterica* comprises 60% of the approximately 2,600 *S. enterica* serovars classified by the O (somatic) antigen and the H (flagellar) antigen (4). Some serovars have a broad host range, while others are adapted to a particular host (5). Human disease largely results from ingestion of contaminated food (vegetables, fruit, and meat) or water (5). Human-adapted typhoid serovars, commonly *Salmonella enterica* serovars Typhi and Paratyphi A, cause typhoid fever, a severe systemic disease, while most nontyphoidal *Salmonella* (NTS) human infections are with broad-host-range serotypes that cause self-limiting gastroenteritis (5). Approximately 5% of all infected patients develop bacteremia, though the rate of systemic infection can be much higher in immunosuppressed patients (6).

NTS infections cause significant morbidity and mortality throughout the world, causing an estimated 94 million illnesses per year, 80 million of which are foodborne (7). In the United States, the Centers for Disease Control and Prevention (CDC) monitor clinical, veterinary, and food sources of *Salmonella* using several surveillance systems that rely on reporting by state and territorial public health laboratories (http://www.cdc.gov/salmonella/index.html). It is estimated that the bacterium is responsible for 1.2 million illnesses each year, including about 23,000 hospitalizations and 400 fatalities (8). Several outbreaks caused by *S. enterica* subsp. *enterica* serovars occur every year. Three NTS serovars, Typhimurium, Enteritidis, and Newport, account for half of the roughly 42,000 laboratory-confirmed cases reported annually by public health laboratories to the CDC (8–10). According to the National Salmonella Surveillance System, the broad-host-range *Salmonella* serovar Typhimurium has been the most common *Salmonella* serovar associated with U.S. outbreaks since 1997.

Despite the burden of *S*. Typhimurium infections on public health systems in the United States and worldwide, relatively little is known about strain diversity (2). Sequenced strains available from public databases (e.g., http://www.ncbi.nlm.nih.gov/genome/152) are known to differ in virulence for inbred mice and antimicrobial resistance phenotypes for example (11–14). Their genomes each contain unique accessory gene repertoires (plasmids, phages, etc.) and sequence variants in core genes (point mutations, insertions, deletions, etc.), of which only some are relevant to these phenotypes. Additionally, there are very diverse sources of *S*. Typhimurium in the United States. Recent outbreaks have been linked to vegetables, nuts, and fruits, in addition to the traditional sources related to food animals (15). Such strains could have significant phenotype diversity and differences in host specificity or colonization linking them to specific environmental sources. Serotyping is based on the antigenic characteristics of lipopolysaccharide (LPS) and flagellin (4); therefore, characterization and definition of specific *S*. Typhimurium lineages through modern DNA sequencing technology could result in improved understanding of strains with greater capability of associating strains with specific food sources and the development of genetic markers that link strains to the sources, allowing outbreaks to be more readily classified and contained (16).

We carried out the present study to assess the diversity of *S*. Typhimurium strains recently isolated in the United States and to place U.S. strains in a global context. We reconstructed the phylogeny for a total of 114 *S*. Typhimurium strains collected between 1946 and 2012 from humans and food animals in the United States and abroad. For a subset of strains with draft or complete genomes, we resolved genetic population structure and assessed genome diversity by examining both gene content and single-nucleotide variants. Finally, we conducted phenotyping assays for genes highlighted by our genetic analyses. Placing a large number of individual strains in an evolutionary context and determining genomic differences among them aids with understanding the genetic basis underlying phenotypic diversity and may assist with the prediction and prevention of future outbreaks.

## RESULTS

### U.S. salmonellosis-associated strains belong to 5 of 10 lineages found in three clades of *S*. Typhimurium that are not restricted by geography, year of acquisition, or host.

To assess the diversity of *S*. Typhimurium strains circulating in the United States and to place those U.S. strains in a global context, we reconstructed phylogeny for 114 *S*. Typhimurium strains collected between 1946 and 2012 from the United States and abroad (see Table S1 in the supplemental material). The U.S. strains were sampled primarily from patients early in the 21st century and from food animals collected during the same time period. Reference strains collected in the United Kingdom, e.g., strains LT2 (17), DT104_NCTC13348 (13), and SL1344 (18), and Malawi, e.g., strain D23580 (19), were included along with strains from Mexico, Japan, and other sub-Saharan African countries.

**FIG 1:**
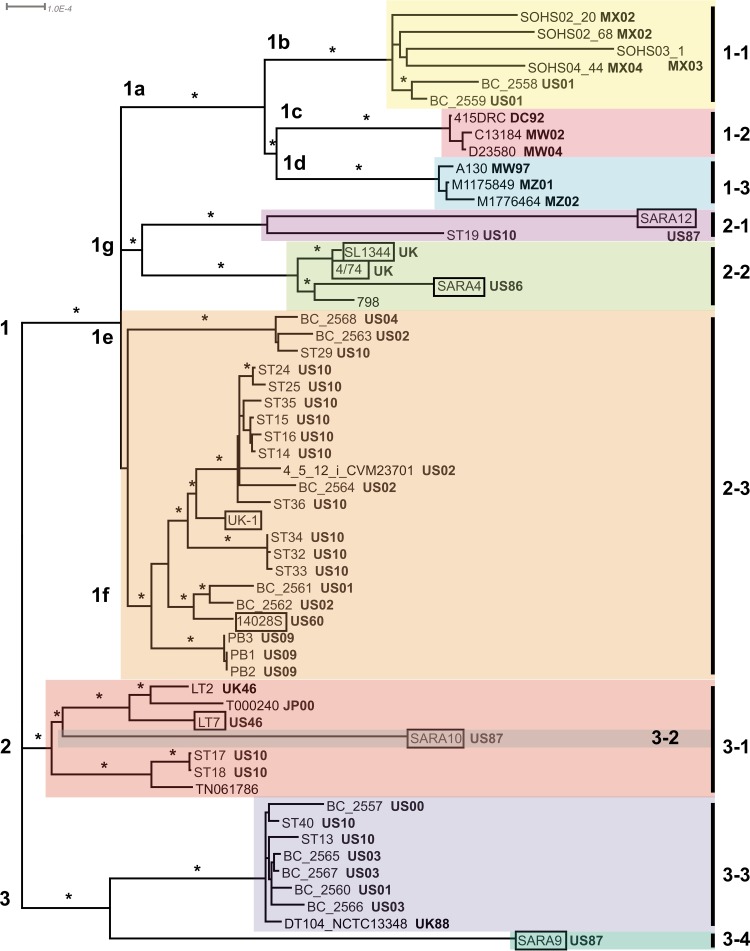
*S*. Typhimurium core gene phylogeny. Maximum likelihood phylogeny of 56 *S*. Typhimurium strains relative to the *S*. Saintpaul SARA23 outgroup. *S*. Saintpaul SARA23 was used to root the tree but was removed in order to view branch topologies among the *S*. Typhimurium strains. The three basal clades are labeled 1 to 3, and seven clade 1 subclades are labeled 1a to 1g. Collection location and year in bold type follow strain names and are abbreviated as follows: DC, Democratic Republic of the Congo; JP, Japan; MW, Malawi; MX, Mexico; MZ, Mozambique; UK, United Kingdom; US, United States. Strains with unknown location and year have no abbreviations. Strains from the CDC collected between 2000 and 2010 are labeled “US10.” Strain names for animal isolates are boxed. BAPS groups are color coded and named in a manner consistent with Table 1. Branches with 100% bootstrap support are labeled with an asterisk.

We first constructed a maximum likelihood tree based on the alignment of nucleotide sequences for the 2,968 core, single-copy genes shared by the 56 *S*. Typhimurium strains with complete or draft genomes and the *S*. Saintpaul SARA23 strain, which represents the closest outgroup to *S*. Typhimurium (20) (see Table S1 and Data Set S1 in the supplemental material). This resulted in a tree comprised of three basal clades (clades 1 to 3), each of which is comprised of one or more “subclades” (Fig. 1). For clarity of discussion, seven subclades of clade 1 are labeled 1a to 1g. Given limited resources to sequence additional strains to a depth required for genome assembly, we then generated a maximum likelihood tree based on k-mer analysis for all 114 *S*. Typhimurium strains using the kSNP software package (21) (see Fig. S1 in the supplemental material). The k-mer tree was based on 6,506 variant positions that were identified in at least 50% of the 114 strains. Though minor differences in topologies exist in some subclades near branch tips, the statistically supported branches in the two trees are consistent.

Because horizontal gene transfer resulting from homologous recombination occurs in bacterial populations and can confound phylogenetic analyses, we complemented our phylogenies with genetic population structure analysis using a nested clustering approach implemented in hierBAPS (hierarchical Bayesian analysis of population structure) (22) and used the program ClonalFrameML (23) to detect recombination in our core gene data set. hierBAPS assigned the *S*. Typhimurium strains to 10 lineages, which generally correspond to well-resolved clades or subclades in our trees (Fig. 1 and Table 1). ClonalFrameML indicated that recombination introduced only a small fraction of substitutions compared to mutation (*r*/*m* = 0.38 where *r* is the relative effect of recombination and *m* is the relative effect of mutation), and strain groupings in the ClonalFrameML tree (see Fig. S2 in the supplemental material) are consistent with those in our core gene phylogeny and resulting from hierBAPS analysis (Fig. 1). Based on these analyses of our data set, at least 10 lineages of *S*. Typhimurium are circulating globally, which are not restricted by geography, year of acquisition, or host.

**TABLE 1:** Association between phylogenetic clades based on core genes, BAPS lineages, and strain traits

Clade	Subclade^a^	BAPS lineage*^b^*	Country of origin^c^	Yr	Host
1	1a	1-1			
		1-2			
		1-3			
	1b	1-1	U.S., Mexico	Multiple	Human
	1c	1-2	Malawi, DR of Congo	Multiple	Human
	1d	1-3	Malawi, Mozambique	Multiple	Human
	1e	2-3	U.S.	Multiple	Human
	1f	2-3	U.S.	Multiple	Human, chicken
	1g	2-1	U.S.	Multiple	Human, equine
		2-2	U.S., U.K.	Unknown	Bovine, rabbit
2		3-1	U.S., U.K., Japan	Multiple	Human, lamb
		3-2	U.S.	1987	Opossum
3		3-3	U.S, U.K.	Multiple	Human
		3-4	U.S.	1987	Parrot

a Subclades 1e and 1f are not distinct BAPS groups.

b BAPS groups 3-2 and 3-4 are comprised of single strains.

c BAPS groups 2-2, 2-3 and 3-1 contain strain(s) of unknown origin. DR of Congo, Democratic Republic of the Congo.

Clade 1 contains the majority of *S*. Typhimurium strains included in this study, grouped into several subclades (Fig. 1; see Fig. S1 in the supplemental material). Subclade 1a is composed of three lineages, BAPS lineage 1-1 includes strains from North America (subclade 1b), and BAPS lineages 1-2 and 1-3 include strains from sub-Saharan Africa (subclades 1c and 1d). In the North American lineage, two U.S. strains, BC_2558 and BC_2559, group with four strains collected in Mexico; all of these strains are multilocus sequence type (MLST) sequence type 302 (ST302) (Table S1). The two sub-Saharan African lineages are comprised of strains with MLST sequence type ST313 and represent those associated with major epidemics in the region during the late 20th and early 21st centuries (24). The topology of the tree indicates that the six North American strains are more closely related to strains from sub-Saharan Africa than to other U.S. strains. This result was intriguing, given the contribution of these African lineages to NTS disease on that continent and the suggestion that these lineages of *S*. Typhimurium may be evolving to cause invasive disease in humans (19, 25).

Subclades 1e and 1f (BAPS lineage 2-3) and 1g (BAPS lineages 2-1 and 2-2) each contain one or more U.S. salmonellosis-associated strains, all of which are ST19 (see Table S1 in the supplemental material). Subclade 1f is divided into additional strongly supported subclades (Fig. 1 and Fig. S1). One of these subclades includes three nearly identical strains, PB1 to PB3, from the 2009 multistate outbreak linked to peanut butter and peanut butter-containing products. Another subclade includes the *Salmonella enterica* serotype 4,5,12:i:− (monophasic variant of *S*. Typhimurium) strain CVM23701 collected in Iowa in 2002 (26, 27). This serotype was the sixth most common *Salmonella* serotype among U.S. cases of human disease in 2006, according to the CDC surveillance summary for that year, and recently the third most common in Europe (28). It has been shown previously to be antigenically and genetically similar to *S*. Typhimurium; for example, strain CVM23701 and many other serotype 4,5,12:i:− strains from the United States and Europe are MLST sequence type ST19. However, strains of this serotype are missing the phase 2 flagellar genes *fljA* (LT2 locus STM2770) and *fljB* (LT2 locus STM2771) and lack expression of the phase 2 flagellar antigen (26). Based on alignment of sequence reads to STM2770 and STM2771, these genes are absent in 11 strains closely related to strain CMV23701: strains ST24, ST25, ST35, ST36, TW16432, TW16687, Eq_0201_630, Eq_0708-22, Eq_0907-73, Ch_0112-54, and Ch_0202-742 (Fig. 1 and Fig. S1). Without *fljA* and *fljB*, these 11 strains are unable to express the phase 2 flagellar antigen and correspond to serotype 4,5,12:i:−. *fljA* and *fljB* are present in all other strains in subclade 1f, which includes the laboratory strains 14028S and UK-1, and subclade 1e (i.e., all other strains in BAPS lineage 2-3).

Nine human isolates and eleven animal isolates collected in the United States are found in clade 2, BAPS lineage 3-1 (Fig. 1; see Fig. S1 in the supplemental material). Though nested within clade 2, the animal strain SARA10 was assigned to a separate lineage by hierBAPS, indicating that this strain is genetically distinct from the others. The LT2 reference strain (17) is located in clade 2 and is the sister taxon to the fluoroquinolone-resistant T000240 strain isolated from a human with gastroenteritis in Japan (29). The relatively high sequence similarity of strain LT2 isolated in the 1940s with strain T000240 isolated in 2000 led Izumiya and colleagues to suggest that progeny of LT2 might be reemerging (29). While their hypothesis is plausible, none of our recent U.S. strains appear to be closely related to either LT2 or T000240.

The previously sequenced definitive type 104 (DT104) NCTC13348 strain (13) is located in clade 3, BAPS lineage 3-3 (Fig. 1; see Fig. S1 in the supplemental material). Seven human strains and ten animal strains collected in the United States are very closely related to strain NCTC13348 based on our analyses. Although the DT104 clone is best known for infecting cattle, this clade includes strains isolated from all animal sources included in our study (Table S1 and Fig. S1).

### *S*. Typhimurium has experienced repeated independent gain and loss of accessory genes.

We assessed genome diversity in *S*. Typhimurium by examining gene content in strains with complete or draft genomes (Fig. 1; see Data Set S2 in the supplemental material). It has been previously shown that gene content varies from strain to strain in *S*. Typhimurium, similar to many other bacterial species (13, 19), and that degradation of common metabolic pathways occurs during host specialization in *Salmonella* serovars (30). Those genes that are variably present across strains comprise the accessory genome. As stated earlier, there are 2,968 core, single-copy genes shared by the 56 *S*. Typhimurium strains with complete or draft genomes and the *S*. Saintpaul SARA23 strain. Excluding *S*. Saintpaul SARA23, the average *S*. Typhimurium genome contains 4,678 genes, and the core genome is comprised of 3,910 genes when pseudogenes were considered present genes (3,428 when pseudogenes were considered absent genes); thus, approximately 16% of the *S*. Typhimurium genomes in our collection are comprised of accessory genes. This number of core genes is consistent with previous studies of the *S. enterica* core and pan-genomes (20, 31). Most accessory genes are acquired by horizontal transfer; thus, *S*. Typhimurium strains that are closely related based on core gene sequence may possess different accessory gene repertoires, while strains that are more distantly related may have accessory genes in common, e.g., they may share a particular prophage or have different prophages that carry subsets of the same genes.

We investigated whether there were clade- or lineage-specific accessory genes by determining the set of genes uniquely interrupted (pseudogenes) or missing, as well as genes uniquely present, in each of clades 1 to 3, and subclades 1a to 1g, and in BAPS lineages that differ from these phylogenetic designations, e.g., lineages 2-1 and 2-2 in subclade 1g. We found that the number of uniquely present and absent genes per clade or lineage is relatively small (see Data Set S3 in the supplemental material). Clade 3 had the largest number of clade-specific interrupted or missing genes (13 total). All human strains and the animal isolate, SARA9, in clade 3 share the previously described loss of 13 allantoin utilization genes (STM0517 to STM0529) (32). Two of these genes (STM0522 and STM0523) have also been lost from strains in subclade 1c, and a third gene (STM0520) has a frameshift in three strains (ST17, ST18, and TN061786) in clade 2; thus, 10 allantoin utilization genes are uniquely lost from clade 3 strains. In addition, there are three clade-specific pseudogenes in these strains: (i) major facilitator superfamily (MFS) xanthosine permease, *xapB* (STM2421); (ii) galactarate dehydratase, *garD* (STM3250); and (iii) a methyl-accepting chemotaxis protein (STM4210). Strains in clade 3 had no uniquely present genes compared to all other strains. When strain SARA9, assigned to a separate lineage (BAPS lineage 3-4), was excluded, the remaining strains in clade 3 share an additional seven uniquely interrupted or missing genes and three uniquely present genes encoding hypothetical proteins (Data Set S3).

BAPS lineage 2-2 in subclade 1g had the largest number of uniquely present genes (16 total), all of which encode proteins annotated as phage related or hypothetical (see Data Set S3 in the supplemental material). Surprisingly, even subclade 1e, including three closely related U.S. strains, had a modest number of uniquely present genes (13 total). In this subclade, strains BC_2563 and BC_2568 have identical gene content, while the ST29 genome contains five genes (annotated as phage related or hypothetical) that are uniquely present compared to all others in the tree. Additionally, these three strains share only one uniquely absent gene, a putative inner membrane protein (homologous to STM2448). When strains in subclades 1e and 1f, comprising BAPS lineage 2-3, are considered together, they share no uniquely present or absent genes.

Given that 16% of the average *S*. Typhimurium genome in our collection is accessory, we expected to find larger numbers of clade- or lineage-specific genes, especially for those including closely related strains from a single geographical region. Yet, all groupings of closely related strains had similarly small numbers of uniquely present and interrupted/missing genes, including subclades 1c and 1d comprised of strains from sub-Saharan Africa. Although we cannot rule out the possibility that paralogous phage genes grouped together in our analysis, this result suggests that while 16% of the genome is accessory, most accessory genes are present (or lost) in more than one lineage. Repeated independent gain and loss suggest that accessory genes may confer a survival or fitness advantage in *S*. Typhimurium.

### Clade-associated variants found in genes involved in host pathogenesis.

We identified single nucleotide polymorphisms (SNPs) in the core gene alignment significantly associated with clades 1 to 3 or subclades 1a to 1g. The results include SNPs that were found in all, or nearly all, members of a given clade and were absent from nonmembers. SNPs fixed in *S*. Typhimurium with respect to the *S*. Saintpaul SARA23 outgroup strain were excluded from this analysis. Of the 3,491 positions that varied among *S*. Typhimurium strains, i.e., those polymorphisms not fixed in *S*. Typhimurium with respect to *S*. Saintpaul, 573 nonsynonymous SNPs (nsSNPs) and 422 synonymous SNPs (sSNPs) were significantly associated with a particular clade (see Data Set S4 in the supplemental material). The vast majority of these SNPs were found in different genes, though there was a small subset of genes with two or three clade-associated polymorphisms.

There were a total of 44, 10, and 91 nsSNPs significantly associated with clades 1, 2, and 3, respectively, and for clade 1 subclades, they ranged from 37 (subclade 1f) to 79 (subclade 1c) and averaged 61. There were 33, 20, and 83 sSNPs significantly associated with clades 1, 2, and 3, respectively, and for clade 1 subclades, they ranged from 18 (subclade 1f) to 55 (subclade 1g) and averaged 41. Some of the clade-associated SNPs were in genes known to be involved in host pathogenesis. Those genes with nsSNPs were of particular interest, since such polymorphisms can affect the function of the encoded protein. Using the “Sorting Intolerant from Tolerant” (SIFT) algorithm (33), we predicted the effects of amino acid changes of all clade-specific nsSNPs found in genes encoding 14 *Salmonella* pathogenicity islands (SPIs), SPI1 and SPI2 effectors, fimbriae, flagella, and additional genes shown to be required for virulence. Several of these nsSNPs were predicted to be nontolerated (see Data Set S4 in the supplemental material). Among these were variants in the SPI2 genes *ttrA*, *ttrC*, and *ttrS*, and in the flavoprotein-encoding *hmpA* gene, all of which we investigated with phenotyping assays.

### Phenotyping assays link gene variants specific to strains in subclade 1a to loss of fitness under nitrosative and oxidative stress.

In our phylogeny, two U.S. strains (BS_2558 and BC_2559) are more closely related to ST313 isolates associated with highly invasive NTS (iNTS) in sub-Saharan Africa than to other U.S. *S*. Typhimurium strains. D23580, a representative iNTS strain from sub-Saharan Africa, has pseudogenes and chromosomal deletions in known virulence-associated genes and in genes that are interrupted or missing from human-adapted *S*. Typhi (19). These observations have led to the hypothesis that *S*. Typhimurium ST313 has adapted to cause invasive disease in humans (6, 19, 24). To investigate the possibility that related U.S. strains are following a similar evolutionary trajectory, we looked for nsSNPs in virulence-associated core genes that cause nontolerated amino acid changes as predicted by the SIFT algorithm.

**FIG 2:**
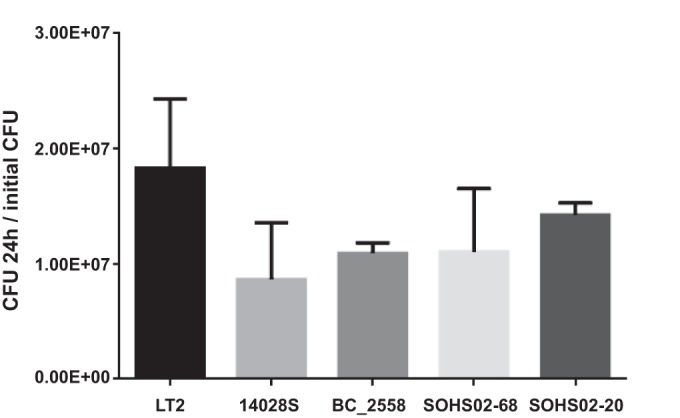
*S*. Typhimurium with nsSNPs in tetrathionate respiration genes show no growth impairment when cultivated anaerobically. Strain LT2 (wild type) and four *S*. Typhimurium strains with amino acid substitutions in TtrC or TtrS were grown anaerobically in BBL tetrathionate broth: strain 14028S (TtrC R187H) and BC_2558, SOHS02-68 and SOHS02-20 (TtrC R187H, TtrS V421G, D204A). The number of CFU were determined at 0 and 24 h. The values are the means plus standard deviations (error bars) of the ratio between the number of CFU at 24 h and the initial CFU count for each strain.

This analysis identified two genes with subclade 1a-specific nsSNPs involved in important pathways for *S*. Typhimurium pathogenesis: the histidine kinase-encoding *ttrS* gene involved in tetrathionate respiration and the nitric oxide (NO)-detoxifying flavohemoglobin-encoding *hmpA* gene. Several *S*. Typhimurium strains contain nsSNPs in genes involved in the tetrathionate respiration pathway (Table 2). Strains in subclade 1a share a nsSNP in *ttrS* (T1262G) resulting in the V421G change predicted to be nontolerated. Additional nontolerated amino acid substitutions in the genes encoding tetrathionate reductase subunit A (*ttrA*) and subunit C (*ttrC*) are observed in members of subclade 1c and clade 1, respectively. The ability to use tetrathionate as an electron acceptor is a key step in the pathogenesis of *Salmonella*, as it provides a growth advantage to the bacterium in the intestine under conditions of inflammation (34). Additionally, the *ttrS* gene is one of several regulators of anaerobic respiration shown to be degraded in host-adapted/host-restricted *Salmonella* serovars (35). To examine whether the amino acid changes observed in TtrA, TtrC, and TtrS have an effect on the growth rate of these isolates, we tested *S*. Typhimurium 14028S, LT2, BC_2558, SOHS02_20 (Fig. 2), and D23580 strains (see Fig. S3 in the supplemental material) in tetrathionate-containing media under anaerobic conditions as described by Winter et al. (34). No significant differences in growth rate were observed between all of the strains tested, suggesting that the nsSNPs observed in *ttrA*, *ttrC*, and *ttrS* in members of subclade 1a are not detrimental to the function of these proteins in the tetrathionate respiration pathway.

**TABLE 2:** Four nsSNPs in tetrathionate respiration genes associated with clades

Clade	Gene	Nucleotide change	Amino acid change	SIFT^a^
1	*ttrC*	G560A	R187H	NT
1a	*ttrS*	T1262G	V421G	NT
1b	*ttrS*	A611C	D204A	T
1c	*ttrA*	T901C	F301L	NT

a Abbreviations: NT, nontolerated; T, tolerated.

**FIG 3:**
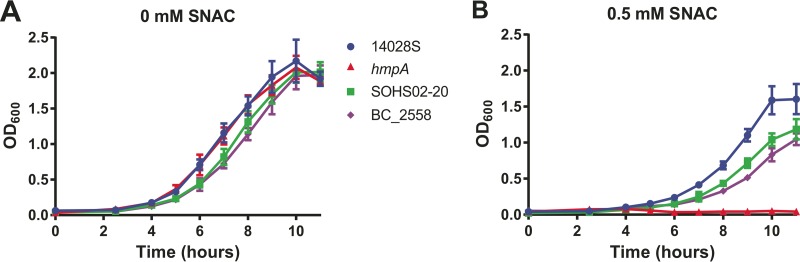
Subclade 1a *S*. Typhimurium with the C667T mutation in HmpA shows impaired growth under nitrosative stress. Growth curves for strains BC_2558 and SOHS02-20, representing subclade 1a strains, strain 14028S (wild type), and the *hmp* null strain (*hmpA* mutant) in the presence and absence of the NO donor, SNAC. The values are means ± standard deviations (error bars) for the three independent trials.

The mechanism to resist nitrosative and oxidative stress generated by host cells is essential for *S*. Typhimurium virulence. Innate immune host cells produce nitric oxide by an inducible NO synthase (iNOS) as an antimicrobial against pathogens (36, 37). In *S*. Typhimurium, the flavohemoprotein HmpA has been shown to be required for virulence in mice (38) and intracellular survival in human macrophages (39). It has also been shown to be a central component in NO detoxification (40, 41). From our genetic variant analysis, *hmpA* was identified as possessing an nsSNP (C667T) in all strains in subclade 1a. This nucleotide change results in an R223C mutation in the protein that is predicted to be nontolerated by the SIFT algorithm. In fact, no amino acid, except for arginine, is predicted to be tolerated at this position. To understand the effect of this substitution in HmpA, we tested the growth rate of strains BC_2558 and SOHS02-20 as representatives of North American strains in subclade 1a, alongside the wild-type 14028S strain and the 14028S *hmpA* null strain (*hmp* mutant) provided by Bang et al. (38) as controls, in the presence or absence of NO donor *S*-nitroso-*N*-acetylcysteine (SNAC). All strains showed equivalent growth rates when grown in media without SNAC; however, in the presence of 0.5 mM SNAC, strains BC_2558 and SOHS02-20 showed intermediate growth defects compared to the wild type and the *hmp* mutant strain (Fig. 3). No nucleotide differences in the noncoding promoter region of *hmpA* were found between strains 14028S, BC_2558, and SOHS02-20. The only nsSNP shared by strains in subclade 1a in genes known to be involved in the nitrosative stress response is in *hmpA*, suggesting that the R223C substitution in HmpA in subclade 1a, although still functional, may result in a less efficient NO-detoxifying enzyme.

**FIG 4:**
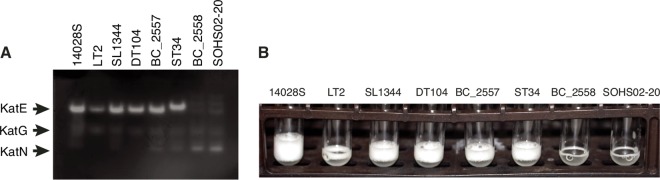
Subclade 1a *S*. Typhimurium with E117G mutation in KatE are deficient in KatE catalase activity. (A) Cellular extracts for eight strains were run individually in a nondenaturing polyacrylamide gel, and the catalase activity was visualized by the method of Woodbury et al. (43). This method relies on the reduction of potassium ferricyanide(III) to potassium ferrocyanide(II) in the presence of hydrogen peroxide, which upon reaction with ferric chloride forms a stable, insoluble Prussian blue pigment. A clear zone is present in regions of the gel that contain catalase activity. (B) Stationary-phase cultures of eight strains were mixed with equal volumes of Triton X-100 and 30% hydrogen peroxide to assay global catalase activity. The formation of oxygen bubbles is apparent in wild-type strains 14028S, LT2, and SL1344 and in strains with the D606G (DT104, BC_2557) and Q45K (ST34) mutations in KatE. Bubble formation is relatively reduced in strains BC_2558 and SOHS02-20 with the E117G mutation.

When looking at variants in U.S. strains BC_2558 and BC_2559, we found an nsSNP in the RpoS-dependent minor catalase gene, *katE*. This gene was not included in our variant analysis of core genes, because it is a pseudogene in strains ST19 and SARA12 in BAPS lineage 2-1 (subclade 1g) due to a premature stop codon (see Data Set S3 in the supplemental material). However, we found that the amino acid substitution in KatE (E117G) is common to all strains in subclade 1a. The E117G mutation has been previously identified in an *S*. Typhimurium blood isolate, which was characterized as peroxide sensitive and was demonstrated to be defective in KatE activity (42). We identified two additional amino acid substitutions in KatE particular to other clades and subclades: (i) clade 3 (D606G) and (ii) a strongly supported subclade within subclade 1f comprised of ST32, ST33, and ST34 (Q45K). Although the only amino acid change predicted to be nontolerated among those observed is E117G, we decided to test one or two representatives of each clade for KatE activity to ascertain whether the other amino acid substitutions were indeed tolerated. The KatE catalase activity was tested in stationary-growth-phase cultures and visualized in nondenaturing polyacrylamide gels by the method of Woodbury et al. (43) (Fig. 4A). In addition, global catalase activity was assayed via the method of Iwase et al. (44) (Fig. 4B). Only the strains from subclade 1a (E117G) showed an absence of KatE activity (Fig. 4A) and decreased catalase activity (Fig. 4B), supporting the predictions of the SIFT algorithm and the previous report of Robbe-Saule et al. (42). To further test the link between the *katE* gene and catalase activity, we generated a *katE* null mutant of strain 14028S. This mutant also showed decreased global catalase activity (Fig. S4). Interestingly, *S*. Typhi and *S*. Paratyphi have several nsSNPs in *katE*. In *S*. Paratyphi A, the gene has several mutations, including E117L that is predicted to be nontolerated by the SIFT algorithm.

## DISCUSSION

*S*. Typhimurium is one of the leading causes of salmonellosis in the United States and worldwide annually, yet very little is known about the diversity of strains that contribute to this major public health concern. Using whole-genome sequencing, we found that there is much diversity among *S*. Typhimurium strains recently isolated in the United States. Phylogenetic and genetic population structure analyses revealed three clades and additional subclades comprised of 10 lineages of *S*. Typhimurium, many of which consist of both U.S. and non-U.S. strains collected in various years spanning the 1940s through the early part of the 21st century. These results indicate that *S*. Typhimurium circulating in the United States is not limited to a single strain or to a set of strains unrelated to those found abroad. Though it is possible that strains isolated in the United States were brought into the country by patients who traveled overseas, this finding is consistent with previous studies of the multidrug-resistant DT104 clone that caused disease worldwide in humans and animals (45). Indeed, clade 3 in our tree contains seven U.S. strains that are closely related to DT104_NCTC13348, which was collected in the United Kingdom in 1988 and sequenced to serve as the reference genome for definitive phage type 104 (DT104) isolates. The topology of our trees is also consistent with a tree generated by Kingsley and colleagues (46) in their study of *S*. Typhimurium DT2, an *S*. Typhimurium variant shown to be host restricted to the feral pigeon. Although their study did not sample any U.S. strains isolated from human salmonellosis cases, reference strains, e.g., LT2, SL1344, etc., and some animal isolates, e.g., SARA4, SARA9, etc., overlap with our study. The three basal clades found in our study are also found in theirs, with the addition of a fourth clade comprised of five strains isolated in the United Kingdom.

Our analysis of genetic population structure further suggest that in the first decade of the 21st century, at least five different lineages of *S*. Typhimurium contributed to human salmonellosis cases in the United States (BAPS lineages 1-1, 2-1, 2-3, 3-1, and 3-3). With the exception of strains associated with peanut products (PB1 to PB3), strains were provided in a blind manner; thus, we are unable to link strains or lineages to specific outbreaks or sporadic events. Nonetheless, our analyses show that each lineage is following a distinct evolutionary trajectory, as illustrated by corresponding accessory gene content and core gene variants.

An unexpected result of our study was the relationship of some U.S. *S*. Typhimurium strains to strains collected in sub-Saharan Africa. Subclade 1a is composed of three lineages, one with strains from the United States and Mexico (BAPS lineage 1-1, subclade 1b) and two with strains from sub-Saharan Africa (BAPS lineages 1-2 and 1-3, subclades 1c and 1d, respectively). The branching of subclades 1b, 1c, and 1d suggest that these lineages may have arisen nearly simultaneously from their common ancestor in the mid-20th century, given estimates for emergence of the two African lineages (47). These U.S. and Mexican strains share many SNPs with the African strains (see Data Set S4 in the supplemental material), as well as 14 of the 23 pseudogenes described as specific to strain D23580 (19). Our phenotyping studies linked nonsynonymous variants specific to subclade 1a to a decreased ability to resist oxidative and nitrosative stress, two strategies used by the human immune system to attack and kill pathogens. Rather than evolving to be more invasive in humans as previously hypothesized, these results suggest that this lineage may be adapting to immunocompromised humans by a loss of pathogenicity factors, such as resistance to oxidative and nitrosative stress, possibly as a result of the worldwide human immunodeficiency virus (HIV) epidemic. HIV infection is strongly associated with recurrent invasive salmonellosis, and defects in macrophage phagocytosis and oxidative burst have been described in HIV-infected individuals (48–51). Circulating and surviving within immune defective individuals therefore may result in the selection of pathogens with a loss of virulence characteristics, since these characteristics may not be as necessary for successful colonization and replication within the host.

Despite intense surveillance by governmental agencies, salmonellosis caused by *S*. Typhimurium occurs every year in the United States. Our study shows that patients can be infected by different *S*. Typhimurium strains, and it provides some initial correlation between observed genotypes and phenotypes*.* Additional research is required to better understand genotype/phenotype correlations and to assign specific strain virulence characteristics to them. Defining simple correlates of pathogenesis may have predictive value for emergence or patient outcome and better equip public health officials when confronted with an outbreak. For instance, subclade 1a strains may be easily determined in a clinical microbiology laboratory by peroxide sensitivity assays on plates. Despite this observation, ultimately genome sequencing should allow more rational classification of microorganisms than the presence of specific surface antigens and biochemical tests.

## MATERIALS AND METHODS

### Bacterial strains.

Details of the 114 *Salmonella enterica* serovar Typhimurium strains investigated in this study are listed in Table S1 in the supplemental material. This includes all *S*. Typhimurium strains received in response to requests for human and animal strains isolated in the United States in the early 21st century, plus publicly available reference strains from other time periods and locations for comparison purposes. Four strains from Mexico and five strains from sub-Saharan Africa were specifically included to expand representation of MLST sequence types ST302 (beyond strains BC_2558 and BC_2559 from Utah) and ST313 (beyond reference genome D23580), respectively; otherwise, there was no selection process to include or exclude *S*. Typhimurium strains from the time period. Genome sequences were generated for 93 strains. Of these strains, 12 strains were recovered from blood samples from bacteremic patients submitted to Utah Public Health Laboratories (UPHL) between 2000 and 2004 (52). Sixteen strains were provided by the Centers for Disease Control and Prevention (CDC) to represent salmonellosis-associated strains with commonly isolated pulsed-field gel electrophoresis (PFGE) patterns between 2000 and 2010. Three strains were isolated by the Washington State Department of Health (WDOH) in association with the 2009 multistate outbreak linked to peanut butter and peanut butter-containing products (http://www.cdc.gov/salmonella/typhimurium/update.html). Fifteen salmonellosis-associated strains from sporadic cases were provided by the Michigan Department of Health and Human Services (MDHHS). Four strains were collected as part of a Mexican multistate surveillance network, in which isolates associated with disease (SOHS02_68 and SOHS04_44) were collected from patients at state referral hospitals, including Hospital General O’Horan (HGO) in Sonora, Mexico, and asymptomatic isolates (SOHS02_20 and SOHS03_1) were collected from feces of kindergarten-age children (53). Forty-three animal isolates were provided by the School of Veterinary Medicine, University of Pennsylvania (PennVet) to represent isolates circulating in U.S. livestock during the same time period as the human strains. The *S*. Saintpaul strain SARA23 was included to represent the closest outgroup to *S*. Typhimurium, based on Fricke et al. (20).

### Genomic DNA extraction and sequencing.

To isolate genomic DNA for sequencing, strains were grown overnight at 37°C with shaking in 3 ml of Luria broth (LB) (BD Biosciences, USA). Genomic DNA was isolated using Gentra Puregene Yeast/Bact. kit (Qiagen, Valencia, CA), according to the manufacturer’s directions. For each genome, either a random-fragment library was constructed using a custom paired-end protocol (54) or standard Illumina Nextera libraries were constructed according to the manufacturer’s guidelines (Illumina Inc., San Diego, CA). Paired-end libraries for each genome were used to generate 76-bp, 100-bp, or 300-bp reads with the Illumina GAIIx and HiSeq 2000 or MiSeq, respectively (coverage for assembled genomes was >150 reads/genomic position). Sequencing of libraries was performed according to the manufacturer’s standards (Illumina Inc., San Diego, CA). 

### Genome assembly and annotation.

Draft genome assemblies were generated for 35 of the 93 sequenced *S*. Typhimurium with Velvet 1.2.10 (55) (see Table S1 and Data Set S2 in the supplemental material). We used ABySS 1.3.4 (56) to generate *de novo* assemblies for an additional 10 *S*. Typhimurium genomes (24) using sequence reads retrieved from the Short Read Archive (SRA) at the National Center for Biotechnology Information (NCBI) (Table S1 and Data Set S2). Homogeneous genome annotation for all strains in this study was derived from automated pipeline prediction and manual curation using the Prokaryotic Genome Analysis Tool (PGAT) (57). The multistrain annotation pipeline mapped the *Salmonella* “pan-genome” (collated from complete *Salmonella* genomes in the PGAT database) into the open reading frames (ORFs) in a six-frame translation of each genome using the protein BLAST tool (58) to identify coding genes and pseudogenes. Pseudogenes were identified by hits that overlap multiple ORFs or were less than 80% of the reference gene length. Novel genes, predicted by Prodigal (59) but not included in the original pan-genome, were also mapped across all strains. Annotation derived from automated prediction was incorporated into the PGAT database and manually curated. False-positive mappings between homologous genes (including mappings to paralogs) were identified by genomic context with the PGAT “synteny view” and corrected using the manual annotation tools. Gene family annotation was based on previously annotated genomes, e.g., strain LT2, D23580, etc., when available, and new gene families were annotated based on searches in the NCBI Conserved Domain Database (CDD) (60). The PGAT database was queried for the orthologous genes belonging to the 56 *S*. Typhimurium complete or draft genomes and the *S*. Saintpaul SARA23 genome included in this study. Single-copy genes that were unambiguously mapped to only one gene in the pan-genome (no close homologs) and were present in all 57 genomes were designated “core” genes. Multiple alignment of core genes with MUSCLE (61) was used to detect variant positions for single nucleotide polymorphism (SNP) analysis. Annotated genomes can be viewed at http://tools.nwrce.org/pgat/.

### Phylogenetic reconstruction.

Phylogeny based on core genes included those orthologous gene families with complete, single-gene copies in each of the 56 *S*. Typhimurium genomes and the *S*. Saintpaul SARA23 genome as determined by PGAT (57). The Presence and Absence function under the PGAT Search Options menu allows users to find genes that are present in all selected genomes for core gene analyses (or present in a selected subset of genomes and absent in a separate subset for gene presence and absence comparisons). The nucleotide sequences of core genes belonging to the same family were aligned using MUSCLE (61), and gene alignments were concatenated to produce complete core gene sets for each genome. The final alignment included 2,968 core genes totaling 2,687,981 nucleotide positions. There were 15,909 total variable positions in the alignment, 3,491 of which were variable among the *S*. Typhimurium strains. The best-fit nucleotide substitution model determined using jModelTest 2.1.4 (62) was the GTR+I+G (general time reversible model with gamma distributed rate variation among sites and I proportion of invariable sites) with shape parameter alpha = 5.63 and the proportion of invariant sites of 0.90. A maximum likelihood tree was constructed with PHYML version 20120412 (63) with four discrete rate categories to approximate the gamma distribution. Base frequencies were estimated from the input sequences. Support for branch nodes was assessed using 100 bootstrap replicates. Phylogeny of 114 *S*. Typhimurium strains was generated using the kSNP software package version 2.1.1, in which SNP discovery was based on k-mer analysis, i.e., single-variant positions within sequences of nucleotide length k (21). The *S*. Saintpaul SARA23 strain was not included, because it increased computation time and space substantially due to the addition of thousands of k-mers unique to it. The maximum likelihood tree was constructed using 31-mers that were identified in at least 50% of the strains and was based on 6,506 SNPs. The 50% requirement provided phylogenetic resolution of the *S*. Typhimurium strains while excluding the SNPs present in only one or a small number of genomes, which are more likely to be the result of sequencing or assembly errors. Support for branch nodes was computed by FastTree version 2.1.3 (64), which is provided in the kSNP package. Tree branches are expressed in terms of changes per total number of SNPs, not changes per site, as SNP-based trees do not include invariant sites. Local support values are based on the Shimodaira-Hasegawa test on the three alternate topologies at each split in the tree. The trees were drawn using Dendroscope version 3.2.10 (65).

### Population genetic analysis.

Population structure was estimated using nested clustering as implemented in hierBAPS (hierarchical Bayesian analysis of population structure) (22). Two nested levels of molecular variation were fitted to the core genome sequence data using 10 independent runs of the optimization algorithm with the maximum number of populations (*K*) varying from 4 to 25 across the runs. The clustering at two levels was consistent across all runs.

### Analysis of recombination.

ClonalFrameML v1.25 was used to detect recombination in the core gene data set. Using the core gene tree, minus *S*. Saintpaul SARA23, as the initial clonal geneology, ClonalFrameML estimated the ratio of recombination and mutation rates, the mean length of recombination events, and average distance between events to be R/Θ = 0.0636, δ = 19.06 bp, and ν = 0.3143, respectively. Given these parameters, the relative effect of recombination and mutation was equal to *r*/*m* = (R/Θ) × δ × ν = 0.38.

### Variant analysis and gene content comparisons.

Custom software scripts were employed to identify variant positions in the core gene alignment of *S*. Typhimurium genomes relative to *S*. Saintpaul SARA23. SNPs that were significantly associated with a clade in the phylogeny (*P* < 0.005) were identified by Fisher’s exact test using R (version 2.14, R Development Core Team 2012, R Foundation for Statistical Computing, Vienna, Austria). Gene content in strains was assessed using the Presence and Absence function in PGAT (57) described under “Phylogenetic reconstruction” (above). The presence of the *fljA* and *fljB* genes was confirmed by alignment of sequence reads using BWA (Burrows-Wheeler aligner) (66) to a single excerpted genome segment of strain LT2 containing *fljA* (STM2770) and *fljB* (STM2771) and 100 bp of flanking genome sequence.

### *S*. Typhimurium growth in the presence of tetrathionate assay.

S. Typhimurium cultures were grown overnight in Luria broth. Overnight cultures were subinoculated in LB and incubated with shaking at 37°C until they reached an optical density at 600 nm (OD_600_) of ~0.2. Bacterial cells were subsequently washed and diluted in 1× phosphate-buffered saline (PBS). BBL tetrathionate broth base supplemented with 6 g/liter of iodine and 5 g/liter of potassium iodine was inoculated with ~100 CFU of each strain and incubated at 37°C for 24 h statically and anaerobically (GasPak system; BD Biosciences). Bacterial numbers were determined by spreading serial 10-fold dilutions on LB plates.

### *S*. Typhimurium growth under nitrosative stress.

Overnight *S*. Typhimurium cultures grown in Luria broth were washed in 1× PBS and normalized to an OD_600_ of 0.05 in M9 minimal medium (BD Biosciences, USA) buffered to pH 5.5 in the presence or absence of 0.5 mM *S*-nitroso-*N*-acetylcysteine (SNAC). SNAC was prepared as previously described (40). Briefly, the *S*-nitrosothiol precursor, *N*-acetyl-l-cysteine (Sigma Aldrich, St. Louis, MO), was dissolved in 1 N HCl and then mixed with sodium-nitrite (water) in equimolar concentrations. The SNAC solution was prepared fresh daily and used immediately. The cultures were grown aerobically at 37°C, and optical density measurements were taken every hour.

### Catalase assays.

To visualize KatE catalase activity, bacterial cells were grown to stationary phase, washed, resuspended in phosphate buffer (50 mM potassium phosphate, 0.1 mM EDTA, pH 7.8) and lysed by sonication. Cell debris was removed by centrifugation at 4°C for 30 min at 18,000 × *g*. The amount of protein in each whole-cell lysate was determined by Coomassie brilliant blue assay. Fifteen micrograms of total protein extracts were loaded on Any kD Mini-PROTEAN TGX (Bio-Rad) nondenaturing polyacrylamide gels, and catalase activity was visualized by the method of Woodbury et al. (43). Additionally, global catalase activity was visualized via evolved oxygen by the method of Iwase et al. (44). Briefly, in clean glass test tubes, 100-µl portions of stationary-phase bacterial cultures resuspended in 1× PBS were mixed with 100 µl of 1% Triton X-100 and 100 µl of 30% hydrogen peroxide. The tubes were gently mixed and incubated at room temperature for 15 min to allow evolved oxygen foam formation.

### Sequence data accession number. 

The sequence data set is available in the Short Read Archive (SRA) repository under BioProject accession no. PRJNA299422.

## SUPPLEMENTAL MATERIAL

Data Set S1  Single-copy core genes used in phylogenetic reconstruction. The locus tags in *S*. Typhimurium strain LT2, gene name, gene description, and coding strand for the 2,968 core genes included in sequence alignments for phylogenetic reconstruction are given. Download Data Set S1, XLSX file, 0.1 MB

Data Set S2 Details of *Salmonella* complete genomes or draft assemblies included in the present study. The genome size and number of contigs, coding genes, and pseudogenes for 56 *S*. Typhimurium strains and 1 *S*. Saintpaul strain are given. Download Data Set S2, XLSX file, 0.03 MB

Data Set S3  The set of genes uniquely interrupted (pseudogenes) or missing, and genes uniquely present, in each of the basal clades and subclades. The locus tag and gene name and description for 23 uniquely present and 35 uniquely interrupted or missing genes are given. The locus tags for genes unique to strains sequenced in this study are from PGAT (http://tools.nwrce.org/pgat/). Download Data Set S3, XLSX file, 0.05 MB

Data Set S4  The 573 nonsynonymous SNPs and 422 synonymous SNPs significantly associated with a basal clade or subclade. Annotation for each SNP, in addition to clade association and SIFT prediction, is shown. Download Data Set S4, XLSX file, 0.1 MB

Figure S1  Maximum likelihood phylogeny of 114 *S*. Typhimurium strains based on k-mers. Animal strains are colored blue. Collection location and year in bold type follow strain names and are abbreviated as follows: DC, Democratic Republic of the Congo; JP, Japan; MW, Malawi; MX, Mexico; MZ, Mozambique; UK, United Kingdom; US, United States. Strains with unknown location and year have no abbreviations. Strains from the CDC collected between 2000 and 2010 are labeled “US10.” The three basal clades are labeled 1 to 3, and seven clade 1 subclades are labeled 1a to 1g. Branches with 100% local support are labeled with an asterisk. Download Figure S1, PDF file, 0.6 MB

Figure S2  ClonalFrameML analysis of recombination in the core gene data set. White vertical bars indicate reconstructed substitutions, and dark blue dots indicate putative recombination events for each branch of the ClonalFrameML tree. Position refers to the position in the final alignment of 2,968 core genes totaling 2,687,981 nucleotide positions. Clades are labeled, and hierBAPS groups are color coded as in Fig. 1. Download Figure S2, PDF file, 0.2 MB

Figure S3  Growth rate of *S*. Typhimurium strains 14028S and D23580 in tetrathionate-containing medium under anaerobic conditions. Strains 14028S (TtrC R187H) and D23580 (TtrC R187H, TtrS V421G, TtrA F301L) show similar growth rates when grown anaerobically in BBL tetrathionate broth. The number of CFU were determined at 0 and 24 h. The values are the means and standard deviations of the ratio between the number of CFU at 24 h and the initial CFU count for each strain. Download Figure S3, DOCX file, 0.1 MB

Figure S4  The 14028S *katE* null mutant is deficient in catalase activity similar to the subclade 1a strains BC_2558 and SOHS02-20. Stationary-phase cultures of four strains were mixed with equal volumes of Triton X-100 and 30% hydrogen peroxide to assay global catalase activity. The formation of oxygen bubbles is apparent in wild-type strain 14028S; however, bubble formation is similarly reduced in the 14028S *katE* null mutant and strains BC_2558 and SOHS02-20 with the E117G mutation. Download Figure S4, DOCX file, 0.1 MB

Table S1  Details of the *Salmonella* strains investigated Table S1, DOCX file, 0.1 MB
