# Supplementary figures and images for: Genomic Analysis of Salmonella enterica Serovar Typhimurium Characterizes Strain Diversity for Recent U.S. Salmonellosis Cases and Identifies Mutations Linked to Loss of Fitness under Nitrosative and Oxidative Stress
Source: mBio. 2016 Mar 8;7(2):e00154-16. doi: 10.1128/mBio.00154-16 (PMC4810482; doi:10.1128/mBio.00154-16)

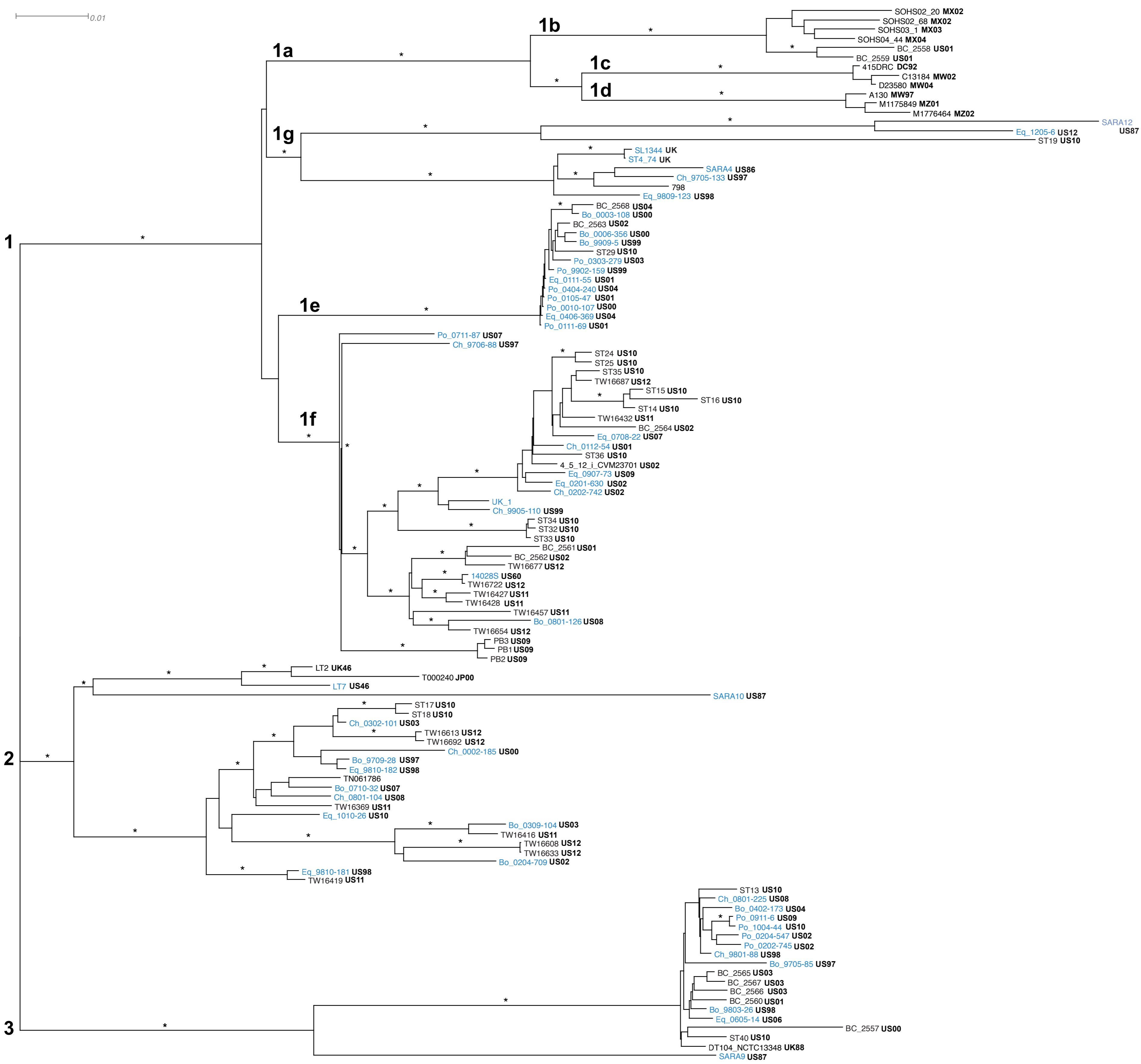

Supplement: Figure S1 — Maximum likelihood phylogeny of 114 S. Typhimurium strains based on k-mers. Animal strains are colored blue. Collection location and year in bold type follow strain names and are abbreviated as follows: DC, Democratic Republic of the Congo; JP, Japan; MW, Malawi; MX, Mexico; MZ, Mozambique; UK, United Kingdom; US, United States. Strains with unknown location and year have no abbreviations. Strains from the CDC collected between 2000 and 2010 are labeled “US10.” The three basal clades are labeled 1 to 3, and seven clade 1 subclades are labeled 1a to 1g. Branches with 100% local support are labeled with an asterisk. Download [file mbo001162710sf1.pdf]

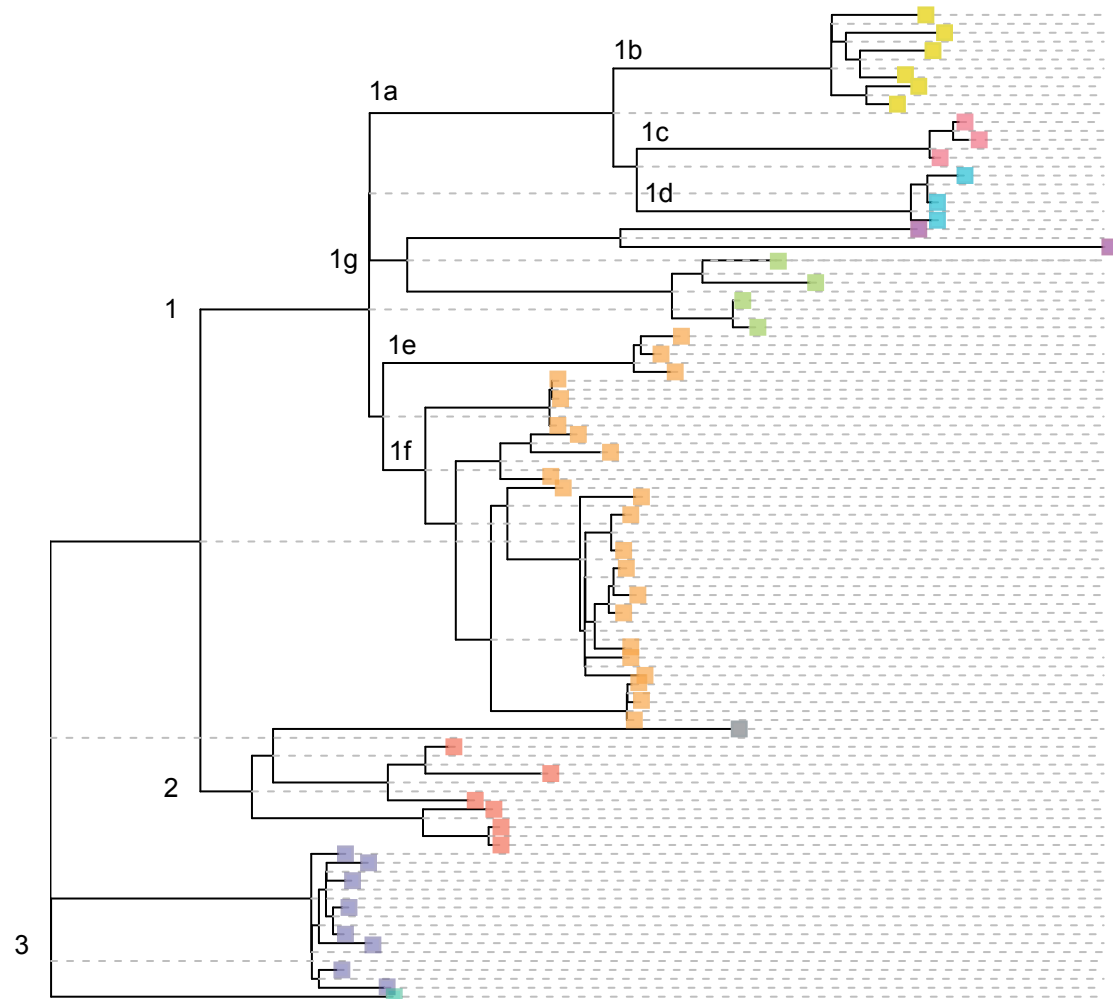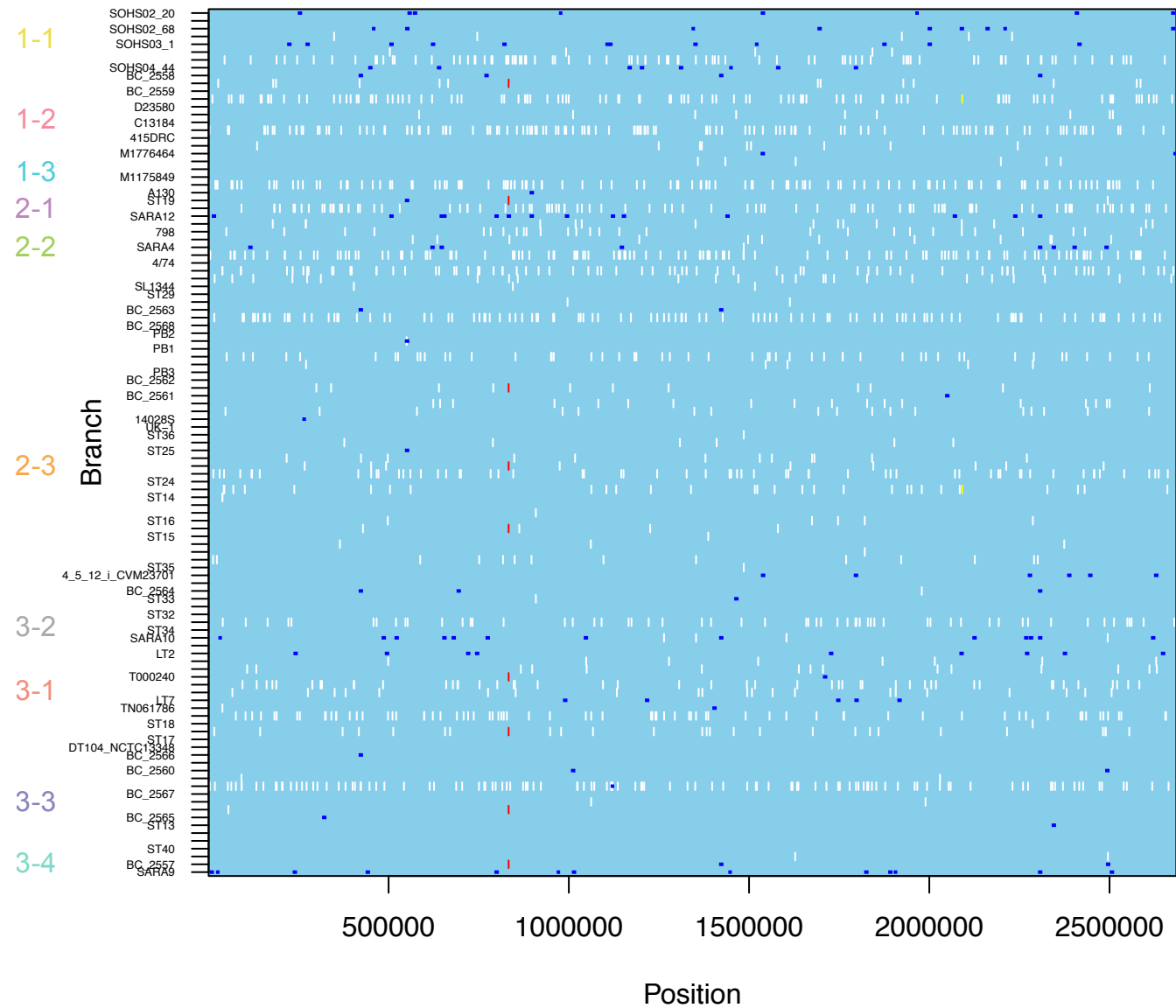

Supplement: Figure S2 — ClonalFrameML analysis of recombination in the core gene data set. White vertical bars indicate reconstructed substitutions, and dark blue dots indicate putative recombination events for each branch of the ClonalFrameML tree. Position refers to the position in the final alignment of 2,968 core genes totaling 2,687,981 nucleotide positions. Clades are labeled, and hierBAPS groups are color coded as in Fig. 1. Download [file mbo001162710sf2.pdf]

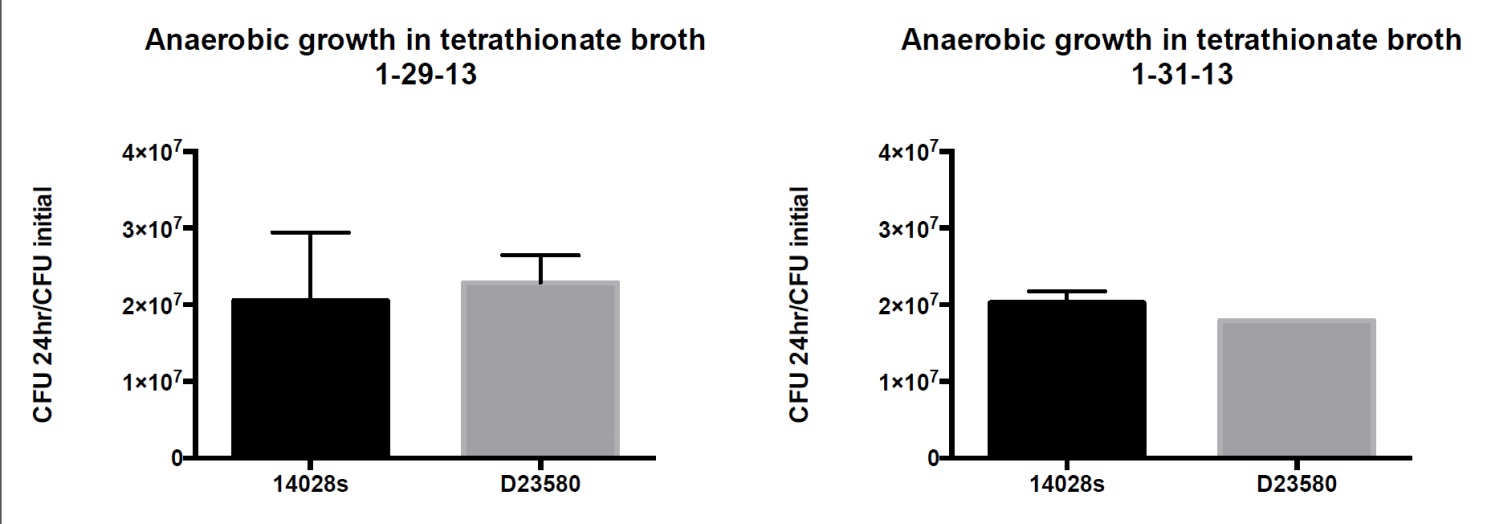

Supplement: Figure S3 — Growth rate of S. Typhimurium strains 14028S and D23580 in tetrathionate-containing medium under anaerobic conditions. Strains 14028S (TtrC R187H) and D23580 (TtrC R187H, TtrS V421G, TtrA F301L) show similar growth rates when grown anaerobically in BBL tetrathionate broth. The number of CFU were determined at 0 and 24 h. The values are the means and standard deviations of the ratio between the number of CFU at 24 h and the initial CFU count for each strain. Download [file mbo001162710sf3.docx]

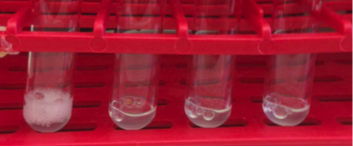


**14028S WT 14028S BC_2558 SOHS02-20**

***katE*::Tet**

Supplement: Figure S4 — The 14028S katE null mutant is deficient in catalase activity similar to the subclade 1a strains BC_2558 and SOHS02-20. Stationary-phase cultures of four strains were mixed with equal volumes of Triton X-100 and 30% hydrogen peroxide to assay global catalase activity. The formation of oxygen bubbles is apparent in wild-type strain 14028S; however, bubble formation is similarly reduced in the 14028S katE null mutant and strains BC_2558 and SOHS02-20 with the E117G mutation. Download [file mbo001162710sf4.docx]
