# Supplementary material for: Genomic Analysis of Salmonella enterica Serovar Typhimurium Characterizes Strain Diversity for Recent U.S. Salmonellosis Cases and Identifies Mutations Linked to Loss of Fitness under Nitrosative and Oxidative Stress
Source: mBio. 2016 Mar 8;7(2):e00154-16. doi: 10.1128/mBio.00154-16 (PMC4810482; doi:10.1128/mBio.00154-16)
Supplement: Table S1 — Details of the Salmonella strains investigated [file mbo001162710st1.docx]

**Table S1. Details of the *Salmonella* strains investigated.**

| **Strain** | **ST** | **Host** | **Host site^a^** | **Yr** | **Location** | **Source** | **Accession** | **Core gene**  **analyses** |
| --- | --- | --- | --- | --- | --- | --- | --- | --- |
| BC_2557 | 19 | Human | Blood | 2000 | Western USA | UPHL | SRS1307952 | Yes |
| BC_2558 | 302 | Human | Blood | 2001 | Western USA | UPHL | SRS1307953 | Yes |
| BC_2559 | 302 | Human | Blood | 2001 | Western USA | UPHL | SRS1307954 | Yes |
| BC_2560 | 19 | Human | Blood | 2001 | Western USA | UPHL | SRS1307955 | Yes |
| BC_2561 | 19 | Human | Blood | 2001 | Western USA | UPHL | SRS1307956 | Yes |
| BC_2562 | 19 | Human | Blood | 2002 | Western USA | UPHL | SRS1307957 | Yes |
| BC_2563 | 19 | Human | Blood | 2002 | Western USA | UPHL | SRS1307958 | Yes |
| BC_2564 | 19 | Human | Blood | 2002 | Western USA | UPHL | SRS1307959 | Yes |
| BC_2565 | 19 | Human | Blood | 2003 | Western USA | UPHL | SRS1307960 | Yes |
| BC_2566 | 19 | Human | Blood | 2003 | Western USA | UPHL | SRS1307961 | Yes |
| BC_2567 | 19 | Human | Blood | 2003 | Western USA | UPHL | SRS1307962 | Yes |
| BC_2568 | 19 | Human | Blood | 2004 | Western USA | UPHL | SRS1307963 | Yes |
| ST13 | 19 | Human | Unknown | 2000-2010 | USA | CDC | SRS1307965 | Yes |
| ST14 | 19 | Human | Unknown | 2000-2010 | USA | CDC | SRS1307966 | Yes |
| ST15 | 19 | Human | Unknown | 2000-2010 | USA | CDC | SRS1307967 | Yes |
| ST16 | 19 | Human | Unknown | 2000-2010 | USA | CDC | SRS1307964 | Yes |
| ST17 | 19 | Human | Unknown | 2000-2010 | USA | CDC | SRS1307968 | Yes |
| ST18 | 19 | Human | Unknown | 2000-2010 | USA | CDC | SRS1307969 | Yes |
| ST19 | 19 | Human | Unknown | 2000-2010 | USA | CDC | SRS1307970 | Yes |
| ST24 | 19 | Human | Unknown | 2000-2010 | USA | CDC | SRS1307971 | Yes |
| ST25 | 19 | Human | Unknown | 2000-2010 | USA | CDC | SRS1307972 | Yes |
| ST29 | 19 | Human | Unknown | 2000-2010 | USA | CDC | SRS1307973 | Yes |
| ST32 | 19 | Human | Unknown | 2000-2010 | USA | CDC | SRS1307974 | Yes |
| ST33 | 19 | Human | Unknown | 2000-2010 | USA | CDC | SRS1307975 | Yes |
| ST34 | 19 | Human | Unknown | 2000-2010 | USA | CDC | SRS1307976 | Yes |
| ST35 | 19 | Human | Unknown | 2000-2010 | USA | CDC | SRS1307977 | Yes |
| ST36 | 19 | Human | Unknown | 2000-2010 | USA | CDC | SRS1307978 | Yes |
| ST40 | 19 | Human | Unknown | 2000-2010 | USA | CDC | SRS1307979 | Yes |
| PB1 | 19 | Human | Unknown | 2009 | WA, USA | WDOH | SRS1307949 | Yes |
| PB2 | 19 | Human | Unknown | 2009 | WA, USA | WDOH | SRS1307950 | Yes |
| PB3 | 19 | Human | Unknown | 2009 | WA, USA | WDOH | SRS1307951 | Yes |
| TW16369 | 19 | Human | Stool | 2011 | MI, USA | MDHHS | SRS1308024 | No |
| TW16416 | 19 | Human | Stool | 2011 | MI, USA | MDHHS | SRS1308033 | No |
| TW16419 | 19 | Human | Stool | 2011 | MI, USA | MDHHS | SRS1308025 | No |
| TW16427 | 19 | Human | Stool | 2011 | MI, USA | MDHHS | SRS1308026 | No |
| TW16428 | 19 | Human | Stool | 2011 | MI, USA | MDHHS | SRS1308034 | No |
| TW16432 | 19 | Human | Stool | 2011 | MI, USA | MDHHS | SRS1308027 | No |
| TW16457 | 19 | Human | Stool | 2011 | MI, USA | MDHHS | SRS1308028 | No |
| TW16608 | 19 | Human | Stool | 2012 | MI, USA | MDHHS | SRS1308035 | No |
| TW16613 | 19 | Human | Stool | 2012 | MI, USA | MDHHS | SRS1308036 | No |
| TW16633 | 19 | Human | Stool | 2012 | MI, USA | MDHHS | SRS1308037 | No |
| TW16654 | 19 | Human | Stool | 2012 | MI, USA | MDHHS | SRS1308038 | No |
| TW16677 | 19 | Human | Stool | 2012 | MI, USA | MDHHS | SRS1308039 | No |
| TW16687 | 19 | Human | Stool | 2012 | MI, USA | MDHHS | SRS1308029 | No |
| TW16692 | 19 | Human | Stool | 2012 | MI, USA | MDHHS | SRS1308040 | No |
| TW16722 | 19 | Human | Stool | 2012 | MI, USA | MDHHS | SRS1308041 | No |
| SOHS02_20 | 302 | Human | Stool | 2002 | Sonora, Mexico | HGO | SRS1307982 | Yes |
| SOHS02_68 | 302 | Human | Blood | 2002 | Sonora, Mexico | HGO | SRS1307981 | Yes |
| SOHS03_1 | 302 | Human | Stool | 2003 | Sonora, Mexico | HGO | SRS1307980 | Yes |
| SOHS04_44 | 302 | Human | Stool | 2004 | Sonora, Mexico | HGO | SRS1307983 | Yes |
| Bo_9705-85 | 19 | Bovine | Unknown | 1997 | PA, USA | PennVet | SRS1308032 | No |
| Bo_9709-28 | 19 | Bovine | Unknown | 1997 | PA, USA | PennVet | SRS1307984 | No |
| Bo_9803-26 | 19 | Bovine | Unknown | 1998 | PA, USA | PennVet | SRS1308005 | No |
| Bo_9909-5 | 19 | Bovine | Unknown | 1999 | PA, USA | PennVet | SRS1308006 | No |
| Bo_0003-108 | 19 | Bovine | Unknown | 2000 | OH, USA | PennVet | SRS1307992 | No |
| Bo_0006-356 | 19 | Bovine | Unknown | 2000 | VA, USA | PennVet | SRS1307993 | No |
| Bo_0204-709 | 19 | Bovine | Unknown | 2002 | PA, USA | PennVet | SRS1307994 | No |
| Bo_0309-104 | 19 | Bovine | Unknown | 2003 | PA, USA | PennVet | SRS1307995 | No |
| Bo_0402-173 | 19 | Bovine | Unknown | 2004 | PA, USA | PennVet | SRS1308030 | No |
| Bo_0710-32 | 19 | Bovine | Unknown | 2007 | PA, USA | PennVet | SRS1308031 | No |
| Bo_0801-126 | 19 | Bovine | Unknown | 2008 | PA, USA | PennVet | SRS1308000 | No |
| Ch_9705-133 | 19 | Chicken | Unknown | 1997 | MD, USA | PennVet | SRS1307985 | No |
| Ch_9706-88 | 19 | Chicken | Unknown | 1997 | MD, USA | PennVet | SRS1307986 | No |
| Ch_9801-88 | 19 | Chicken | Unknown | 1998 | MD, USA | PennVet | SRS1308007 | No |
| Ch_9905-110 | 19 | Chicken | Unknown | 1999 | GA, USA | PennVet | SRS1307989 | No |
| Ch_0002-185 | 19 | Chicken | Unknown | 2000 | MO, USA | PennVet | SRS1307988 | No |
| Ch_0112-54 | 19 | Chicken | Unknown | 2001 | TX, USA | PennVet | SRS1308001 | No |
| Ch_0202-742 | 19 | Chicken | Unknown | 2002 | PA, USA | PennVet | SRS1308002 | No |
| Ch_0302-101 | 19 | Chicken | Unknown | 2003 | TX, USA | PennVet | SRS1308003 | No |
| Ch_0801-104 | 19 | Chicken | Unknown | 2008 | TN, USA | PennVet | SRS1308004 | No |
| Ch_0801-225 | 19 | Chicken | Unknown | 2008 | MD, USA | PennVet | SRS1308019 | No |
| Eq_9809-123 | 19 | Equine | Unknown | 1998 | USA | PennVet | SRS1307990 | No |
| Eq_9810-181 | 19 | Equine | Unknown | 1998 | USA | PennVet | SRS1307991 | No |
| Eq_9810-182 | 19 | Equine | Unknown | 1998 | USA | PennVet | SRS1308016 | No |
| Eq_0111-55 | 19 | Equine | Unknown | 2001 | USA | PennVet | SRS1308008 | No |
| Eq_0201-630 | 19 | Equine | Unknown | 2002 | USA | PennVet | SRS1308015 | No |
| Eq_0406-369 | 19 | Equine | Unknown | 2004 | USA | PennVet | SRS1307996 | No |
| Eq_0605-14 | 19 | Equine | Unknown | 2006 | KT, USA | PennVet | SRS1307997 | No |
| Eq_0708-22 | 19 | Equine | Unknown | 2007 | USA | PennVet | SRS1307998 | No |
| Eq_0907-73 | 19 | Equine | Unknown | 2009 | USA | PennVet | SRS1308020 | No |
| Eq_1010-26 | 19 | Equine | Unknown | 2010 | USA | PennVet | SRS1308021 | No |
| Eq_1205-6 | 19 | Equine | Unknown | 2012 | OK, USA | PennVet | SRS1308022 | No |
| Po_9902-159 | 19 | Porcine | Unknown | 1999 | PA, USA | PennVet | SRS1308018 | No |
| Po_0010-107 | 19 | Porcine | Unknown | 2000 | NC, USA | PennVet | SRS1307987 | No |
| Po_0105-47 | 19 | Porcine | Unknown | 2001 | NC, USA | PennVet | SRS1308009 | No |
| Po_0111-69 | 19 | Porcine | Unknown | 2001 | PA, USA | PennVet | SRS1308017 | No |
| Po_0202-745 | 19 | Porcine | Unknown | 2002 | PA, USA | PennVet | SRS1307999 | No |
| Po_0204-547 | 19 | Porcine | Unknown | 2002 | PA, USA | PennVet | SRS1308010 | No |
| Po_0303-279 | 19 | Porcine | Unknown | 2003 | PA, USA | PennVet | SRS1308011 | No |
| Po_0404-240 | 19 | Porcine | Unknown | 2004 | IL, USA | PennVet | SRS1308012 | No |
| Po_0711-87 | 19 | Porcine | Unknown | 2007 | PA, USA | PennVet | SRS1308013 | No |
| Po_0911-6 | 19 | Porcine | Unknown | 2009 | PA, USA | PennVet | SRS1308014 | No |
| Po_1004-44 | 19 | Porcine | Unknown | 2010 | PA, USA | PennVet | SRS1308023 | No |
| LT7 | 19 | Lamb | Unknown | 1946 | CO, USA | Okoro et al. 2012 | ERS007493 | Yes |
| SARA4 | 19 | Rabbit | Unknown | 1986 | IN, USA | Okoro et al. 2012 | ERS007502 | Yes |
| SARA9 | 98 | Parrot | Unknown | 1987 | CA, USA | Okoro et al. 2012 | ERS007507 | Yes |
| SARA10 | 19 | Opposum | Unknown | 1987 | CA, USA | Okoro et al. 2012 | ERS007508 | Yes |
| SARA12 | 19 | Equine | Unknown | 1987 | LA, USA | Okoro et al. 2012 | ERS007510 | Yes |
| A130 | 313 | Human^P^ | Blood | 1997 | Malawi | Okoro et al. 2012 | ERS007468 | Yes |
| M1776464 | 313 | Human | Blood | 2002 | Mozambique | Okoro et al. 2012 | ERS007624 | Yes |
| M1175849 | 313 | Human | Blood | 2001 | Mozambique | Okoro et al. 2012 | ERS009051 | Yes |
| C13184 | 313 | Human^P^ | Blood | 2002 | Malawi | Okoro et al. 2012 | ERS007471 | Yes |
| 415DRC | 313 | Human | Blood | 1992 | DR of Congo | Okoro et al. 2012 | ERS009039 | Yes |
| LT2 | 19 | Human | Unknown | 1946 | United Kingdom | McClelland et al. 2001 | NC_003197.1 | Yes |
| T000240 | 19 | Human | Stool | 2000 | Japan | Izumiya et al. 2011 | NC_016860.1 | Yes |
| DT104_NCTC13348 | 19 | Human | Stool | 1988 | United Kingdom | Cooke et al. 2008 | NC_022569.1 | Yes |
| TN061786 | 19 | Unknown | Unknown | Unknown | Unknown | NCBI | NZ_AERV00000000.1 | Yes |
| D23580 | 313 | Human^P^ | Blood | 2004 | Malawi | Kingsley et al. 2009 | NC_016854.1 | Yes |
| 4_5_12_i_CVM23701 | 19 | Human | Unknown | 2002 | IA, USA | Soyer et al. 2009 | NZ_ABAO00000000.1 | Yes |
| 14028S | 19 | Chicken | Heart, liver | 1960 | Unknown | Jarvik et al. 2010 | NC_016856.1 | Yes |
| SL1344 | 19 | Bovine | Bowel | Unknown | United Kingdom | Kröger et al. 2012 | NC_016810.1 | Yes |
| ST4/74 | 19 | Bovine | Bowel | Unknown | United Kingdom | NCBI | NC_016857.1 | Yes |
| UK-1 | 19 | Horse | Unknown | 1991 | Unknown | Luo et al. 2011 | NC_016863.1 | Yes |
| 798 | 19 | Unknown | Unknown | Unknown | Unknown | NCBI | NC_017046.1 | Yes |
| Saintpaul_SARA23 | NA | Human | Unknown | Unknown | PA, USA | NCBI | NZ_ABAM00000000.2 | Yes |

^a^ Patient HIV status for three sub-Saharan Africa strains was reported as positive (P) by Okoro et al. (2012). The patient HIV status for other human isolates is unknown.
